# Supplementary material for: PROMO: an interactive tool for analyzing clinically-labeled multi-omic cancer datasets
Source: BMC Bioinformatics. 2019 Dec 26;20:732. doi: 10.1186/s12859-019-3142-5 (PMC6933892; doi:10.1186/s12859-019-3142-5)
Supplement: Supplementary file 1 — Additional file 1: Figure S1. Clustering Panel. Figure S2. Biomarker Discovery. Table S1. List of differentially expressed genes. Figure S3. Label Management Panel. Figure S4. Multi-omic sample clustering. [file 12859_2019_3142_MOESM1_ESM.docx]

**Supplementary Material**


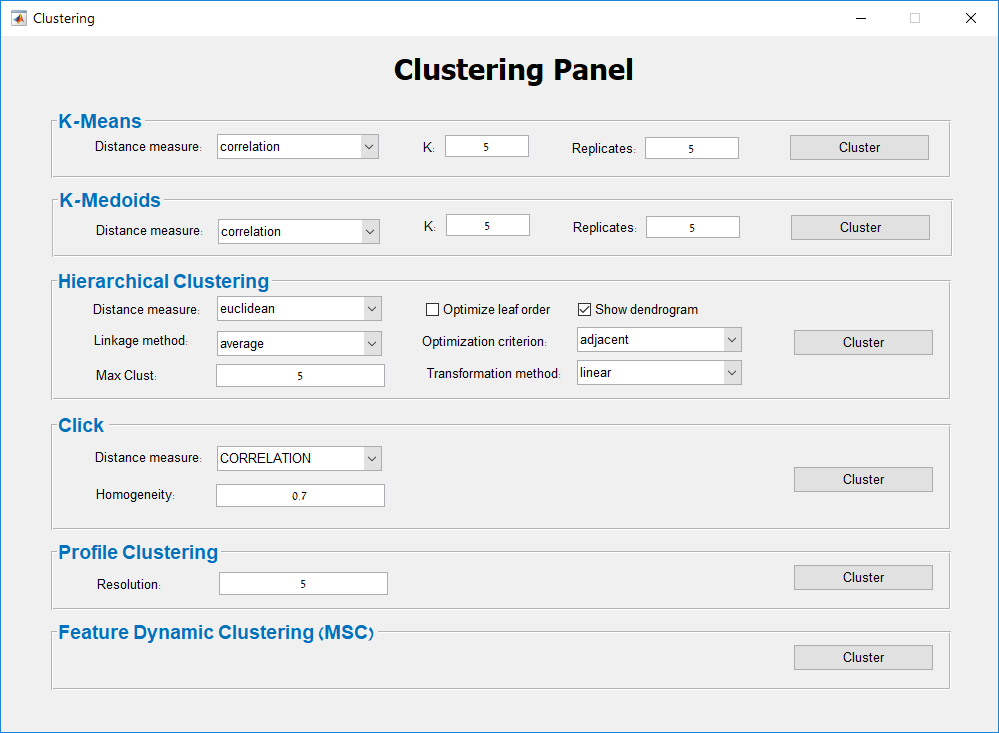

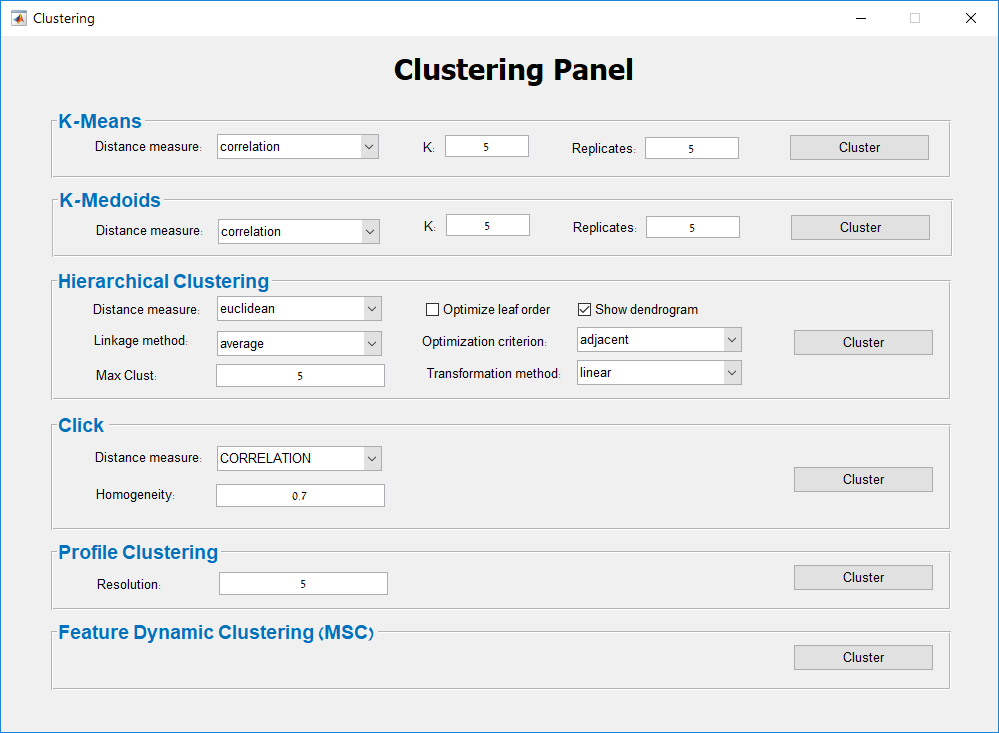


**Figure S1: Clustering Panel** The clustering panel allows the selection of a clustering algorithm and its relevant parameters. Clustering can be applied both on samples and on genes. The resulting clusters are added as a new sample label and can be explored on PROMO's main screen with respect to any other clinical label (See Figure 3).


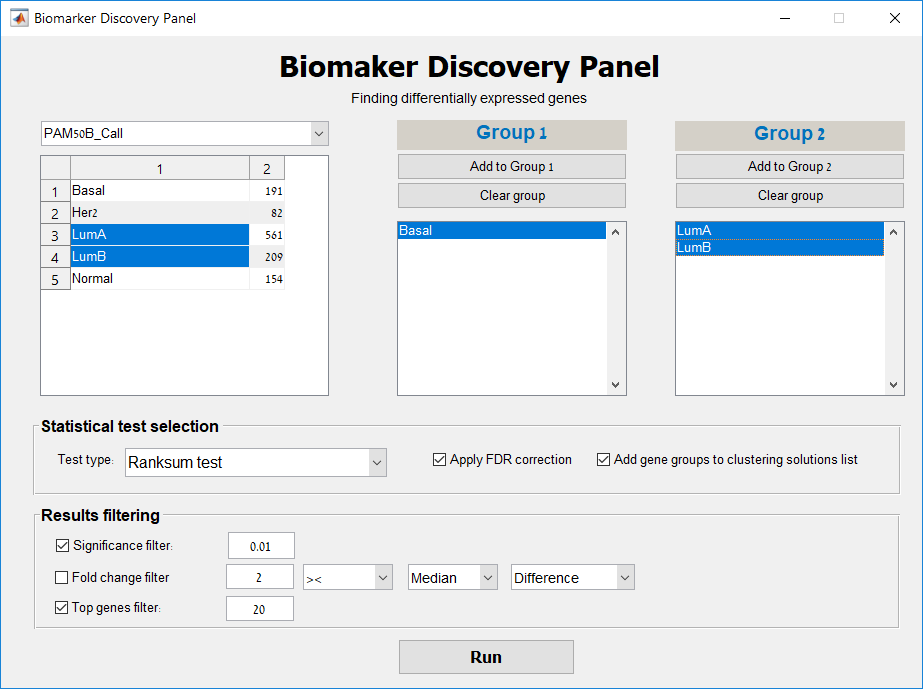


**A**

**B**

**Figure S2: Biomarker Discovery** (A) This panel is used for identifying genes that are differentially expressed between sample groups defined by any sample label. Statistical tests vary by the label types, and include t-test, Ranksum test, ANOVA and Kruskal-Wallis. After optional filtering, the resulting list of genes is saved to a file sorted by p-value. Here two groups were defined, according to the PAM50 label. One group corresponds to the basal and the other to the LumA and Lum B categories. See Table S1 for the resulting set of differentially expressed genes. (B) The feature patterns of the identified genes are presented on PROMO's main screen together with any selected sample labels. Here we see the expression levels of the 20 genes that were identified by the test in A, after row normalization).

| id | Gene Symbol | p-value (Test: Ranksum test on PAM50B_Call [Basal](n=191) vs. [LumA,LumB](n=770)) | Fold Change | p-value Rank |
| --- | --- | --- | --- | --- |
| 1 | CXorf61 | 2.33E-123 | 4.5604 | 1 |
| 2 | LEMD1 | 1.72E-122 | 3.1005 | 2 |
| 3 | ART3 | 3.17E-118 | 5.294 | 3 |
| 4 | HORMAD1 | 1.75E-113 | 5.8894 | 4 |
| 5 | GABBR2 | 7.04E-111 | 4.2383 | 5 |
| 6 | SLC26A9 | 4.21E-101 | 2.4335 | 6 |
| 7 | OPRK1 | 2.22E-99 | 2.5534 | 7 |
| 8 | GATA3 | 3.27E-99 | -4.02715 | 8 |
| 9 | CCKBR | 5.86E-99 | 2.1373 | 9 |
| 10 | ROPN1 | 8.44E-99 | 6.1879 | 10 |
| 11 | MLPH | 5.43E-98 | -5.1038 | 11 |
| 12 | ESR1 | 7.07E-98 | -7.07625 | 12 |
| 13 | SLC39A6 | 9.68E-98 | -2.7197 | 13 |
| 14 | FOXA1 | 3.21E-97 | -6.7128 | 14 |
| 15 | TBC1D9 | 6.71E-97 | -4.06355 | 15 |
| 16 | LOC145837 | 2.99E-96 | -4.18775 | 16 |
| 17 | CT62 | 3.12E-96 | -3.65845 | 17 |
| 18 | RASGEF1C | 5.50E-96 | 2.1274 | 18 |
| 19 | AGR3 | 1.12E-95 | -9.2111 | 19 |
| 20 | FOXC1 | 1.63E-95 | 4.26835 | 20 |

**Table S1: List of differentially expressed genes.** The 20 genes with the most significant differential expression between the groups defined in Figure S2A are shown. Genes are sorted by their Ranksum test p-values. Genes with positive fold change are over-expressed on the Basal samples compared with the Luminal samples. Here, for instance, we see that the Estrogen Receptor gene (ESR1) is ranked 12^th^ and exhibits a significant under-expression on the Basal tumors samples (the Triple-Negative subtype) compared to the Luminal tumor samples.


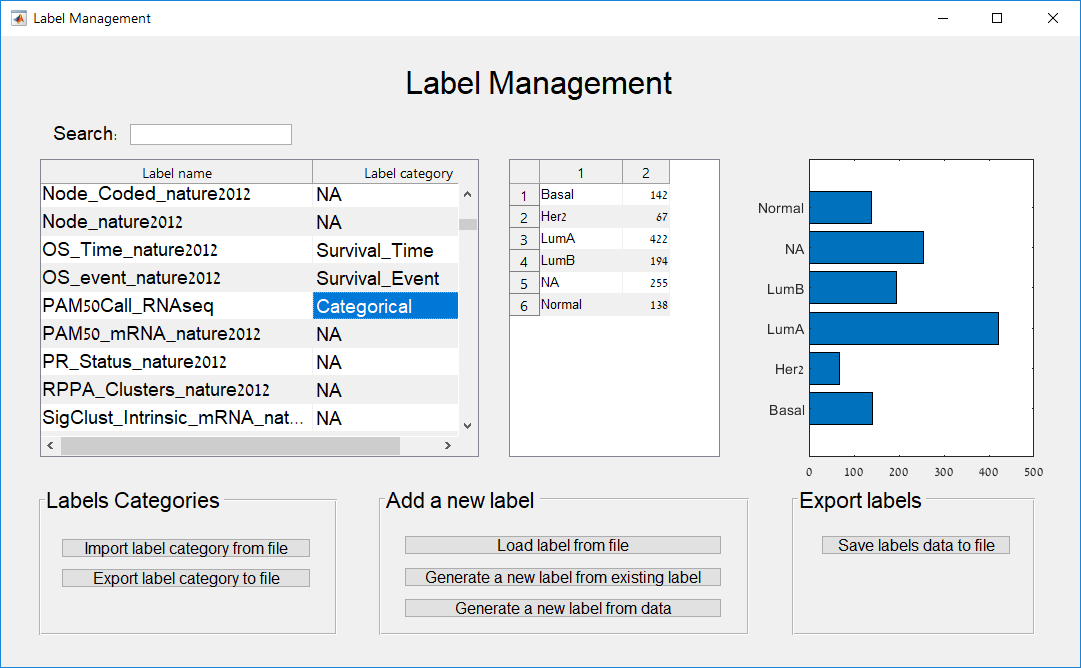


**Figure S3:** **Label Management Panel** This panel allows the management of sample labels, including removing, renaming and viewing the distribution of values of a label. Labels can be assigned to category types, and those types determine the statistical test that can be used for calculating their enrichment on sample clusters. Both labels and their categories can be loaded and saved to files. New labels can be generated from existing labels (by uniting label values for instance), or from genomic data (e.g., translating expression values of selected gene to LOW/HIGH labels). Lastly, the distribution of values for the selected label is displayed as an histogram on the right.


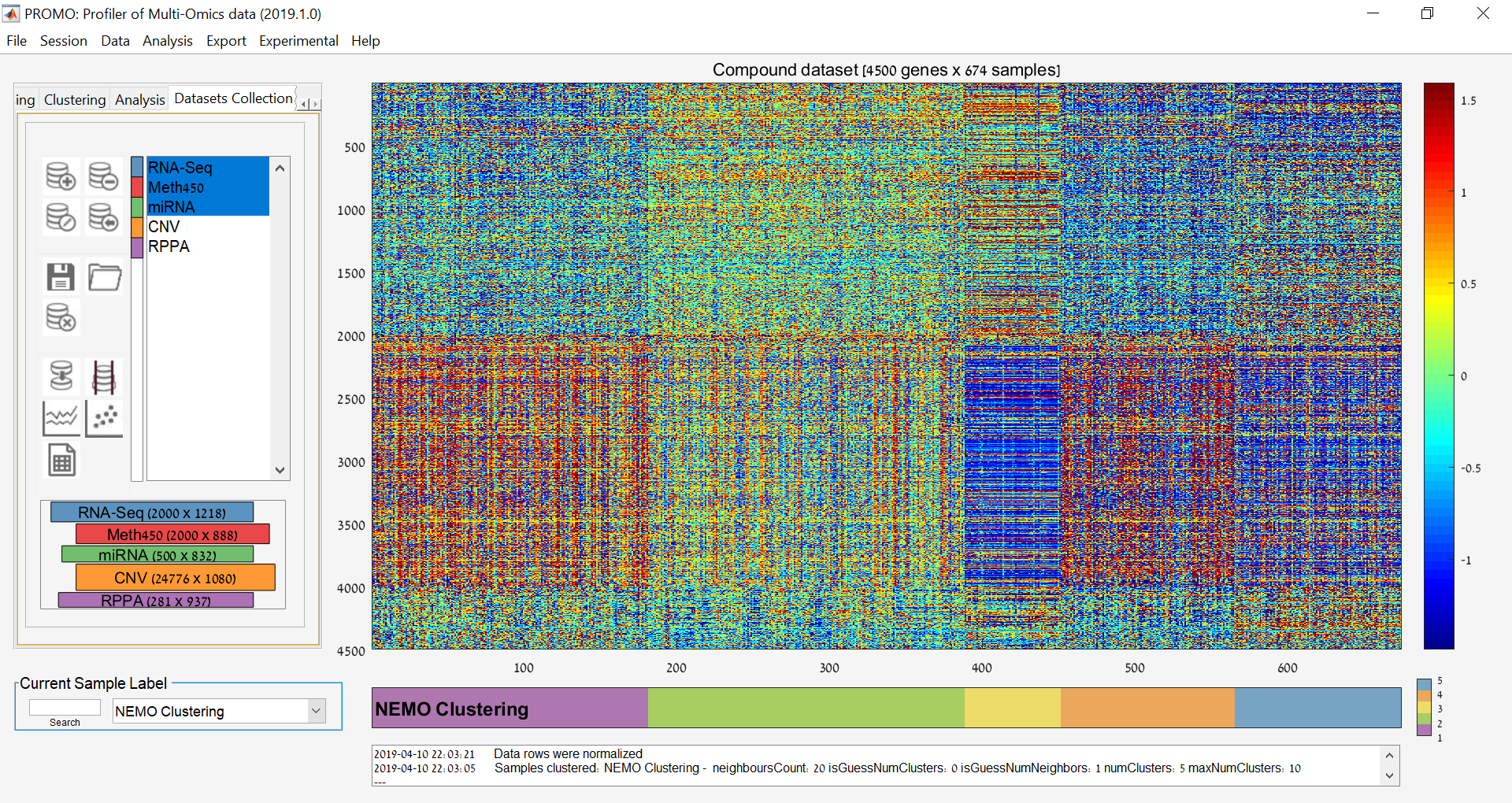


**C**

**B**

**A**

**Figure S4:** **Multi-omic sample clustering** Screenshot of PROMO's main screen after applying multi-omic clustering on 674 breast tumor samples from TCGA. The 'Dataset Collection' panel on the left was used to select the three omics to be used in the clustering. Here features from three different omics were used: (A) RNA-Seq (2000 features), (B) DNA methylation arrays (2000 features) and (C) miRNA arrays (500 features). Algorithm NEMO [39] was applied on the subset of samples appearing in the three omics into 5 groups, shown on the label bar below the matrix. The genomic matrix displays concatenation of the 4500 features included in the analysis after row normalization, with samples grouped by cluster. The 1^st^ and 4^th^ clusters from the left have high methylation signals, while the second and third have higher gene expression signals. Clustering of tumor samples using a multi-omic algorithms integrates data from different biological levels and thus has the potential of revealing biological regulatory patterns that are missed in single omic analysis.
